# Gamma-Ray Sterilization Effects in Silica Nanoparticles/γ-APTES Nanocomposite-Based pH-Sensitive Polysilicon Wire Sensors

**DOI:** 10.3390/s110908769

**Published:** 2011-09-13

**Authors:** Jing-Jenn Lin, Po-Yen Hsu

**Affiliations:** 1 Department of Applied Materials and Optoelectronic Engineering, National Chi Nan University, Puli, Nantou, 54561, Taiwan; 2 Department of Electrical Engineering, National Chi Nan University, Puli, Nantou, 54561, Taiwan; E-Mail: rummel.hsu@gmail.com

**Keywords:** 3-aminopropyltriethoxysilane, polydimethylsiloxane, silica nanoparticles, γ-ray sterilization, post-sterilization UV annealing

## Abstract

In this paper, we report the γ-ray sterilization effects in pH-sensitive polysilicon wire (PSW) sensors using a mixture of 3-aminopropyltriethoxysilane (γ-APTES) and polydimethylsiloxane (PDMS)-treated hydrophobic fumed silica nanoparticles (NPs) as a sensing membrane. pH analyses showed that the γ-ray irradiation-induced sensitivity degradation of the PSW pH sensor covered with γ-APTES/silica NPs nanocomposite (γ-APTES+NPs) could be restored to a condition even better than prior to γ-ray irradiation by 40-min of post-sterilization room-temperature UV annealing. We found that the trapping charges caused by γ-ray sterilization primarily concentrated in the native oxide layer for the pH sensor covered with γ-APTES, but accumulated in the γ-APTES+NPs layer for the γ-APTES+NPs-covered sensor. It is believed that mixing the PDMS-treated silica NPs into γ-APTES provides many γ-APTES/SiO_2_ interfaces for the accumulation of trapping charges and for post-sterilization UV oxidation, thus restoring γ-ray-induced sensor degradation. The PDMS-treated silica NPs not only enhance the sensitivity of the pH-sensitive PSW sensors but are also able to withstand the two-step sterilization resulting from γ-ray and UV irradiations. This investigation suggests γ-ray irradiation could be used as a highly-efficient sterilization method for γ-APTES-based pH-sensitive biosensors.

## 1. Introduction

Medical devices, especially implantable biosensors, need to be sterilized to eliminate potential microbial contamination from sources such as bacterial spores, fungi and viruses [1,2]. Conventionally, sterilization is accomplished through high- and low-temperature techniques. High-temperature techniques include steam sterilization (100 °C or 121 °C) or hot-air sterilization (180 °C) [3,4]. Although moist heat sterilization offers both cost effectiveness and established efficacy, some biosensors with reference electrodes cannot withstand temperatures above 100 °C or invasive moisture [5]. Low-temperature sterilization techniques include ethylene oxide (EO) gas aeration (20–50 °C) [6], plasma sterilization [7,8], UV illumination [9,10], and γ-ray or e-beam irradiation [11]. EO gas aeration provides effective disinfection but is highly toxic [1]. The assurance of surface inactivation makes non-thermal plasma a highly-effective method of sterilization, but microbial inactivation is a slow process, and some biosensors are highly sensitive to plasma [8]. γ-rays or e-beams are able to penetrate through materials, thus providing very efficient disinfection, but both can seriously degrade the devices being sterilized.

Among these sterilization methods, γ-ray irradiation provides the greatest sterilization efficiency and penetration. A standard γ-ray dosage of 25 kGy, corresponding to CE-Marking regulations for the distribution of medical products in the European Union [1], achieves effective disinfection against even large amounts of highly-resistant microorganisms [12,13]. However, the ionizing radiation can damage chemosensors or organic biomaterial plane-covered biosensors, and how to best restore this damage is still an important issue. Preventing ionizing irradiation-induced damage is a major challenge in γ-ray sterilization applications.

The effects of high-energy ionizing radiation have been a concern for at least two decades. Techniques for developing radiation-hardness in silicon-based [14,15] or compound semiconductor electronic devices [16] are well established. For silicon-based electronic devices, radiation-induced damage primarily accumulates in the oxide near the SiO_2_/Si interface. Although the ionizing radiation-induced charge traps can be easily removed by a 400 °C, N_2_ anneal, restoring γ-ray sterilization-induced damage to most ion sensitive field effect transistor (ISFET) chemosensors or organic biomaterial plane-covered sensors at such high temperature is impractical. In this paper, we propose a method to restore γ-ray sterilization-induced damage to γ-APTES-covered PSW pH sensors. Through introducing PDMS-treated silica NPs into the γ-APTES, the γ-ray sterilization induced-damage to the PSW can be restored by room temperature UV annealing.

## 2. Experimental Section

**Figure 1.**
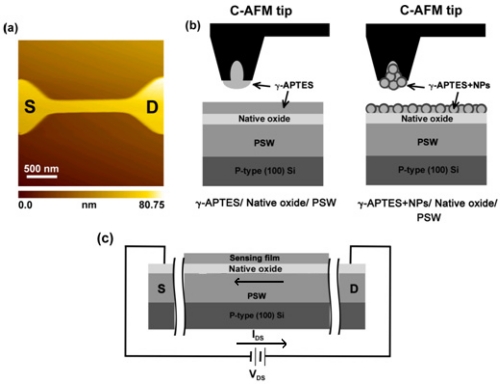
**(a)** AFM image of the PSW sensor, **(b)** schematic diagrams of the γ-APTES and γ-APTES+NPs coated onto the PSW sensor surface using an FIB processed C-AFM tip, and **(c)** schematic of the PSW sensor structure and electrical measurements.

pH-sensitive PSW sensors were fabricated on a 30 nm-thick SiO_2_ coated p-type (100) silicon wafer. The poly-Si layer was doped with phosphorous and had a thickness of 80 nm and a sheet resistance of 40–50 Ω/□. An optical lithography process was used to define the PSW pattern on the poly-Si layer. Reactive-ion etching was then used to fabricate the PSW sensors with a fixed channel length of 3 μm and a line width of 358 nm. Figure 1(a) shows the AFM images of the PSW. The γ-APTES solution was prepared through an ethanol solution containing 1% pure anhydrous γ-APTES. Some of the prepared γ-APTES solution was added by PDMS-treated hydrophobic fumed silica NPs (R202, Evonik Degussa GmbH, Germany) to form the γ-APTES+NPs solution. The mixed weight ratio of the γ-APTES solution and NPs was 100:1 and the average primary silica particle size was 14 nm. The mixture was subjected to ultrasonic vibration for 10 min to disperse the silica NPs. A focus-ion-beam (FIB) processed capillary atomic-force-microscope (C-AFM) tip was used to assist in the membrane coating and solution transferrance. Figure 1(b) shows the schematic diagram for the C-AFM tip-coating process. A SEIKO 300 HV AFM system was used, and details of the process can be found in our previous reports [17,18]. Previous studies have obtained a uniform thickness of 21–23 nm for the C-AFM tip-coated membranes [19]. The RSD of the 100 thicknesses for the C-AFM tip-coating was 5.33%. The PSWs were divided into three batches, with the first batch coated with γ-APTES (*i.e.*, γ-APTES/native-oxide/PSW structure), the second coated with γ-APTES+NPs (*i.e.*, γ-APTES+NPs/native-oxide/PSW structure), and the third left uncoated (*i.e.*, native-oxide/PSW structure) for comparison. After coating with a layer of γ-APTES or γ-APTES+NPs, the first and second batches were cured on a hotplate at 120 °C for 5 min. The samples were then subjected to γ-ray irradiation with a total dose of 25 kGy. The UV (λ = 365 nm) annealing was performed in open air at room temperature.

The damage caused by γ-ray sterilization to the PSW sensor and its subsequent recovery were tested through pH analysis using standard phosphate buffer solution (1× PBS, 140 mM NaCl, 10 mM phosphate buffer, and 3 mM KCl) with pH values from 3 to 10 (Merck Inc.). The tested phosphate buffer solutions were coated onto the PSW surface with the FIB processed C-AFM tip, similar to the process used in the coating of the γ-APTES or γ-APTES+NPs mixture. Time-dependent sensitivity analysis of the pH detections were performed for seven consecutive days following the γ-ray irradiation. All currents flowing through the PSW channel before/after γ-ray irradiation, UV annealing, and pH analyses were measured by a semiconductor parameter analyzer (Agilent 4156C). Figure 1(c) presents a schematic of the PSW sensor structure and electrical measurement.

## 3. Results and Discussion

**Figure 2.**
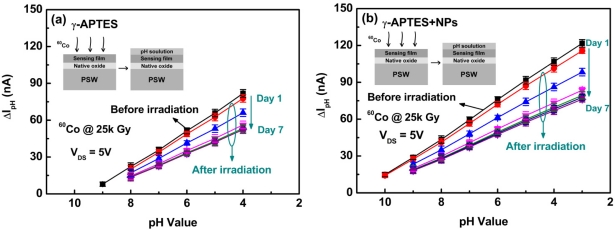
Channel current change ΔI_pH_ as a function of pH value over time, before and after γ-ray irradiation for the **(a)** γ-APTES/native-oxide/PSW and **(b)** γ-APTES+NPs/native-oxide/PSW pH sensors.

Figure 2(a,b) shows the channel current change ΔI_pH_ as a function of pH values before and after γ-ray irradiation for the γ-APTES/native-oxide/PSW and γ-APTES+NPs/native-oxide/PSW pH sensors, respectively. The ΔI_pH_ is defined as Δ*I*_pH_ = *I*(after coating pH solution) – *I*(before coating pH solution). The detection principle is based on the field effect of the PSW on the channel conductivity modulation. The hydrogen ions can easily bind with NH_2_ bonds on the γ-APTES surface, forming NH_3_^+^ bonds, and the surface-charge state will be altered when pH solutions of different concentrations are coated onto the surface of the PSW sensor, thus changing the conductivity of the PSW channel and the current flowing through the PSW channel. As shown in Figure 2(a,b), following γ-ray irradiation, the sensitivity of both the γ-APTES/native-oxide/PSW and the γ-APTES+NPs/native-oxide/PSW sensors degraded over the first three days at an accelerated rate. The rate of degradation then slowed between the fourth and fifth days, and finally stopped after the sixth day.

**Figure 3.**
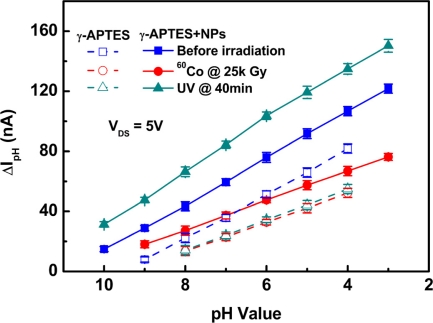
pH analyses of the γ-APTES/native-oxide/PSW and γ-APTES + NPs/native-oxide/PSW pH-sensitive sensors before and after γ-ray sterilization, and after γ-ray + (40-min UV) treatments.

Figure 3 shows the pH analyses of the γ-APTES/native-oxide/PSW and the γ-APTES+NPs/native-oxide/PSW sensors, showing conditions pre-sterilization, post-sterilization, and post-UV annealing. The post-sterilization curves shown in Figure 3 were obtained from the stable values of the seventh day in Figure 2.

As shown in Figure 3, for the pre-sterilization pH-sensitive γ-APTES/native-oxide/PSW sensor, the pH detection range was from about pH 4 to pH 9 and the sensitivity was 12 nA ± 0.03 nA/pH unit. For the pre-sterilization γ-APTES+NPs/native-oxide/PSW, the pH detection range was from about pH 3 to pH 10 and the sensitivity was 13.6 nA ± 0.02 nA/pH unit. After sterilization, for the γ-APTES-coated pH-sensitive sensor, the detection range degraded to between pH 4 to pH 8 and the sensitivity was about 10.5 nA ± 0.02 nA/pH unit. For the post-sterilization γ-APTES+NPs-coated sensors, the detection range degraded to between pH 3 to pH 9 and the sensitivity was about 11.9 nA ± 0.03 nA/pH unit. It was clear that the sensitivity and pH detection range were seriously degraded both for the γ-APTES/native-oxide/PSW and γ-APTES+NPs/native-oxide/PSW sensors after γ-ray irradiation. However, following a 40-min post-sterilization UV anneal, the detection range of the γ-APTES+NPs/native-oxide/PSW sensors reverted back to between pH 3 to pH 10 and the sensitivity was restored to 16.5 nA ± 0.04 nA/pH unit. However, for the γ-APTES/native-oxide/PSW sensor, post-sterilization UV annealing resulted in no discernible change in the detection range and sensitivity. The sensitivity degradation induced by γ-ray sterilization in the pH-sensitive γ-APTES+NPs/native-oxide/PSW sensor could be restored by the UV anneal back to levels even superior to those before the sterilization, but the UV anneal had nearly no effect on the γ-APTES/native-oxide/PSW pH sensor. It is reported that PDMS can be oxidized by exposure to UV illumination [20,21]. In our recent work, we also found that the Si-O-Si absorption peak of PDMS-treated silica NPs in FTIR analysis increased with UV exposure time [22]. We thus believe that part of the methyl group on the NPs surface is oxidized by UV irradiation, and the broken Si-O-Si bonds at the γ-APTES/NPs interfaces are possibly restored during re-oxidation, resulting in reduced radiation-induced positive trap charges. As the positive trap charges are reduced, the hydrogen ions will be more easily absorbed on the membrane surface, thus restoring sensitivity. In addition, the alignment of the γ-APTES NH_2_ bonds will be enhanced by UV illumination [23,24], which likely also contributes to the improvement of sensitivity.

**Figure 4.**
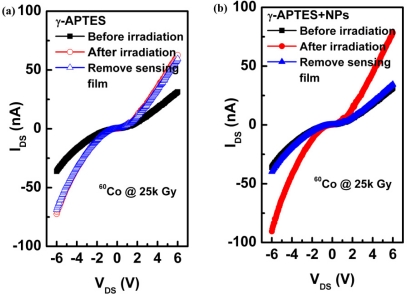
Channel current I_DS_ as a function of drain-to-source voltage V_DS_ before and after γ-ray sterilization, and the removal of the post-irradiated sensing films for the **(a)** γ-APTES/native-oxide/PSW **(b)** γ-APTES+NPs/native-oxide/PSW sensors.

To determine the effect of γ-ray irradiation on the pH-sensitive PSW sensors, the current change ΔI_DS_ was measured without coating the pH solutions onto the sensors. Figure 4(a) shows the channel current I_DS_ as a function of drain-to-source voltage V_DS_ before and after γ-ray sterilization for the γ-APTES/native-oxide/PSW sensors without the tested pH buffer solutions. After measuring the I_DS_-V_DS_ with γ-ray irradiation, the γ-APTES/native-oxide/PSW was soaked in D.I. water to hydrolyze the post-irradiated γ-APTES membrane. The I_DS_-V_DS_ was then obtained again, as shown in Figure 4(a), which shows the I_DS_ increases after γ-ray irradiation and is nearly unchanged following the removal of the post-irradiated γ-APTES sensing film. The same procedures were performed for the γ-APTES+NPs/native-oxide/PSW device, with results shown in Figure 4(b) which shows the I_DS_ increases significantly following γ-ray irradiation, but then returns to its original pre-irradiation value following the removal of the post-irradiated γ-APTES+NPs membrane. Figure 4(a,b) shows the γ-ray-induced charge traps primarily accumulated in the native oxide layer for γ-APTES/native-oxide/PSW sensor, but concentrated in the γ-APTES+NPs layer for γ-APTES+NPs/native-oxide/PSW device. No previous reports could be found on this phenomenon.

**Figure 5.**
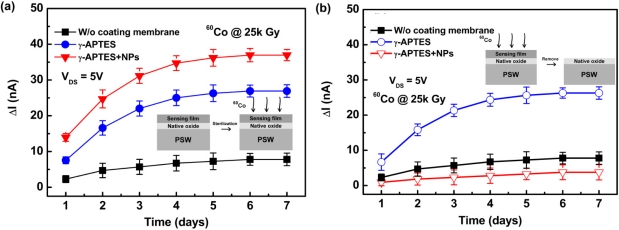
Channel current change ΔI_DS_ *versus* time curves, **(a)** after γ-ray sterilization for the native-oxide/PSW, γ-APTES/native-oxide/PSW and γ-APTES+NPs/native-oxide/PSW sensors, **(b)** after removal of the post-irradiated γ-APTES or γ-APTES + NPs membranes. The γ-ray irradiated native-oxide/PSW without membrane coating shown in (a) is re-plotted for comparison.

Figure 5(a) shows the channel current change ΔI_DS_ as a function of time following γ-ray sterilization for the γ-APTES/native-oxide/PSW and γ-APTES+NPs/native-oxide/PSW sensors. The control sample of the native-oxide/PSW sensor is also depicted for comparison. At this stage, the channel current change ΔI_DS_ is defined as Δ*I*_DS_ = *I*_DS_(after sterilization) – *I*_DS_(before sterilization). Figure 5(a) shows the ΔI_DS_ induced by γ-ray sterilization increases significantly in both the γ-APTES/native-oxide/PSW and γ-APTES+NPs/native-oxide/PSW sensors as compared to the control sample (*i.e.*, native-oxide/PSW sensor). Moreover, the ΔI_DS_ increased with time at an accelerated rate over the first three days following γ-ray sterilization, and then slowed between the fourth and fifth days, before finally stopping after the sixth day. High energy irradiation can break the Si-O-Si bonds near the SiO_2_/Si interface in silicon-based electronic devices, thus forming fixed oxide charges or interface charge trapping states [13,14]. Several studies have reported that the γ-APTES ethoxy group OEt can react with Si-OH to form Si-O-Si bonds on the oxide surface following the curing process [25,26]. Thus it is reasonable to expect that γ-ray sterilization would break the Si-O-Si bonds in the γ-APTES/SiO_2_ interface. Given the large number of γ-APTES/SiO_2_ interfaces in the γ-APTES+NPs membrane provided by the high surface-to-volume ratio of the NPs, it was expected that γ-ray sterilization would induce more charge traps as shown in Figure 5(a). Moreover, because the ΔI_DS_ increased over time following γ-ray sterilization, possible bond transformations and defect migrations to the γ-APTES/SiO_2_ interfaces were expected to occur in the γ-APTES or γ-APTES+NPs membranes following γ-ray sterilization, thus forming charge trapping states.

To further study this charge-trapping behavior, two batches of γ-APTES/native-oxide/PSW and γ-APTES+NPs/native-oxide/PSW were prepared for γ-ray irradiation with seven samples in each batch. On the first day following irradiation, one sample from each batch was selected and soaked in D.I. water to hydrolyze the post-irradiated γ-APTES or γ-APTES+NPs membranes on the native-oxide/PSW. Following the removal of the post-irradiated γ-APTES or γ-APTES+NPs membranes, the I_DS_ of the resulting native-oxide/PSW structures were measured, and the ΔI_DS_ obtained was compared to the I_DS_ prior to the removal of the irradiated membranes. Using the same procedures, the remaining sensors were selected and processed in the six days following the γ-ray irradiation. Figure 5(b) shows the ΔI_DS_ *versus* time curves after γ-ray sterilization and following the removal of the post-irradiated γ-APTES or γ-APTES+NPs membranes. For comparison, Figure 5(b) shows the re-plotting of the γ-ray irradiated control sample of native-oxide/PSW without the membrane coating from Figure 5(a). The ΔI_DS_ curve was nearly unchanged for the γ-APTES/native-oxide/PSW following the removal of the post-irradiated γ-APTES membrane, but was greatly reduced for the γ-APTES+NPs/native-oxide/PSW following the removal of the post-irradiated γ-APTES+NPs film. The ΔI_DS_ curve of the resulting native-oxide/PSW obtained by removing the γ-APTES+NPs membrane from the post-irradiated γ-APTES+NPs/native-oxide/PSW is even lower than that of the γ-ray irradiated control sample of native-oxide/PSW without the membrane coating. The addition of silica NPs in γ-APTES seems to keep most of the γ-ray induced trap charges inside the membrane, preventing them from migrating to the γ-APTES/native-oxide interface. Comparison of Figure 5(a,b) shows that the γ-ray induced trap charges mostly accumulated in the native oxide layer of the γ-APTES/native-oxide/PSW sensors, but mostly accumulated in the γ-APTES+NPs layer for the γ-APTES+NPs/native-oxide/PSW sensors, identical to the phenomena seen in Figure 4(a,b).

**Figure 6.**
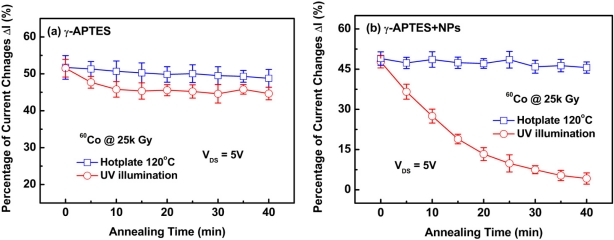
Percentage of current change ΔI_DS_% *versus* thermal or UV annealing time curves after 25 kGy γ-ray irradiation for the **(a)** γ-APTES/native-oxide/PSW and **(b)** γ-APTES+NPs/native-oxide/PSW sensors.

Since a slight rise in sensor temperature was observed during post-sterilization UV annealing, the recovery of radiation-induced damage was possibly due to annealing out by heat. Thus we should make comparisons to determine the relative effects of UV and thermal annealing on damage recovery. Both the γ-ray irradiated γ-APTES/native-oxide/PSW and the γ-APTES+NPs/native-oxide/PSW sensors were subjected to 120 °C thermal annealing (γ-ray+T) or UV annealing (γ-ray+UV). Figure 6(a,b) shows the curves for the percentage of current changes ΔI_DS_% *versus* annealing time with γ-ray+T or γ-ray+UV treatments for the γ-APTES/native-oxide/PSW and the γ-APTES+NPs/native-oxide/PSW sensors, respectively. The annealing treatments were performed on the seventh day following γ-ray irradiation while the sensor states remained stable. Figure 6(a) shows that neither 120 °C heat treatment nor room temperature UV irradiation could restore the damage incurred by the γ-APTES/native-oxide/PSW sensor. Meanwhile, as observed in Figure 6(b), thermal treatment had no restorative effect on the membrane modified by PDMS-treated SiO_2_ NPs, but the 40-min UV anneal was found to completely restore the damage.

**Figure 7.**
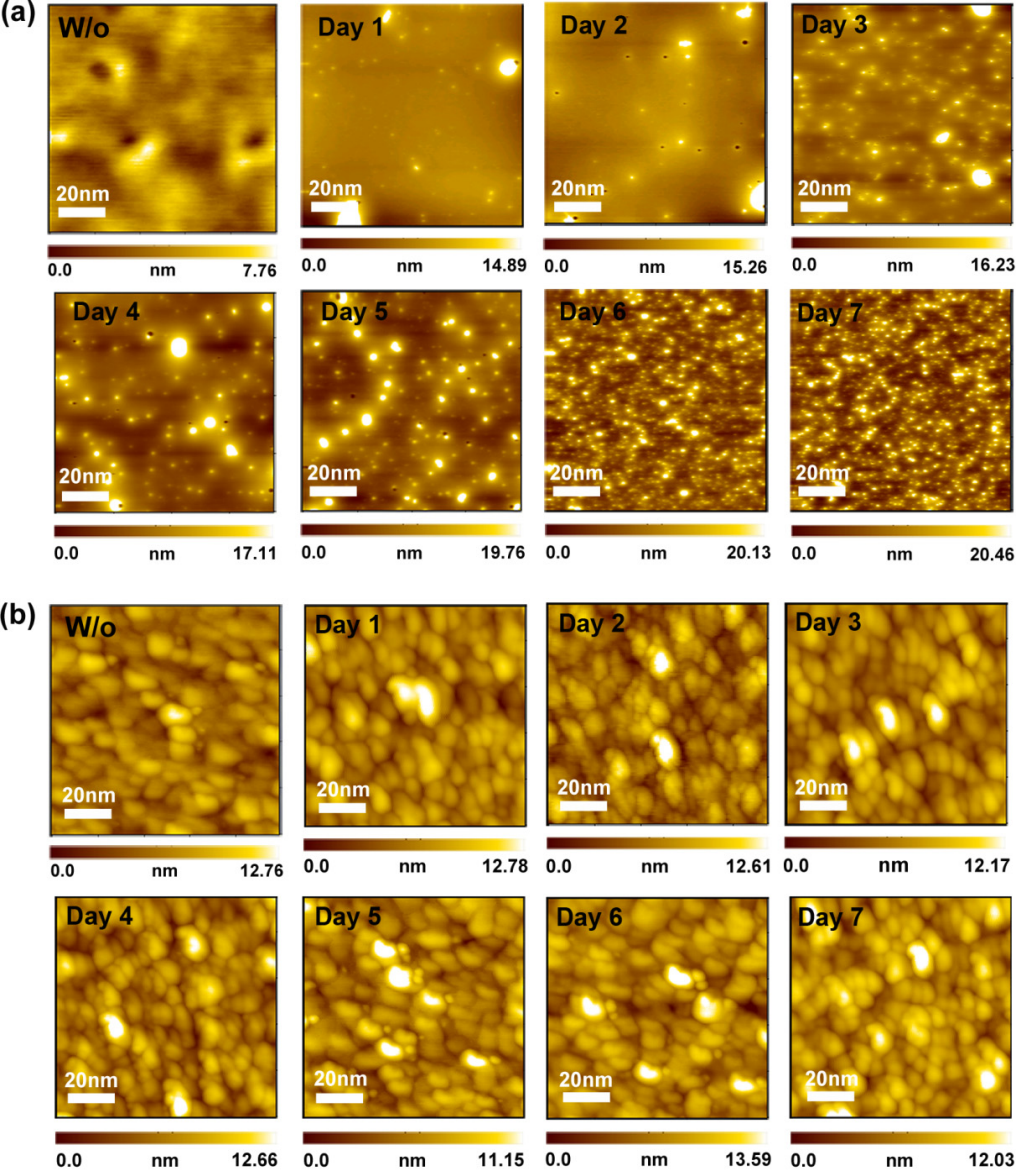
Time-dependent AFM surface morphology images of the pre- and post-sterilization pH-sensitive PSW sensors coated with **(a)** γ-APTES, and **(b)** γ-APTES+NPs.

Figure 7(a) shows the pre- and post-sterilization AFM surface morphology images of the pH-sensitive PSW sensors covered with γ-APTES. Before sterilization, no obvious bright spots were observed in the AFM surface morphology. However, after sterilization, scattered bright spots were found in the AFM images. The bright spot variations are believed to be related with the radiation-induced trap charge aggregations. They are not only time-dependent in number but also in size and shape. Qualitatively, the number of these bright spots grew quickly over the first 3 days following irradiation, and then slowed in the 4th and 5th days, and finally stopped growing after the 6th day. This is consistent with the change in ΔI_DS_ over time, as observed in Figure 5(a). It has been proven that the radiation-induced bright spots observed by non-contact AFM result from charge accumulation within the oxide [27,28]. We thus believe that the changes in the ΔI_DS_ over time shown in Figure 5(a) can be attributed to the time-dependency of charge accumulations as observed in Figure 7(a).

Figure 7(b) shows the AFM surface morphology images of the pre- and post-sterilization for the pH-sensitive PSW sensors covered with γ-APTES+NPs. Although few bright spots were observed in the AFM images following irradiation, the bright spots shows growth trends similar to that seen in Figure 7(a). As seen in Figure 5(a), the channel current change ΔI_DS_ of the γ-APTES+NPs/native-oxide/PSW sensor is larger than that for the γ-APTES/native-oxide/PSW sensor, and thus it would be expected to induce more trap charges following irradiation. It is thus believed that the trap charges might be distributed in the cluster between the silica NPs or in-between the NPs layers, which were not detected by AFM.

**Figure 8.**
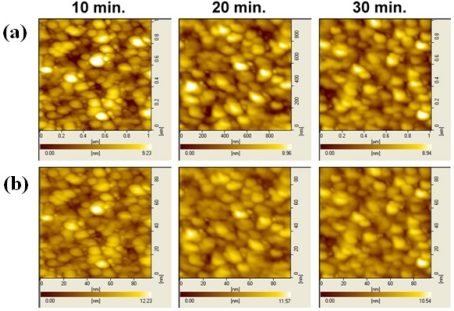
Annealing time-dependent AFM surface morphology images of **(a)** thermal annealing, and **(b)** UV annealing of the pH-sensitive PSW sensors coated with γ-APTES+NPs.

Figure 8(a,b) shows the AFM surface morphology images of the γ-APTES+NPs annealed by post-sterilization thermal and UV treatments, respectively. It is clear that the bright spots are not affected by the 120 °C thermal treatment with different annealing times, but increased UV annealing time reduced the number of bright spots, and they tend to disappear entirely after 40-min UV annealing.

**Figure 9.**
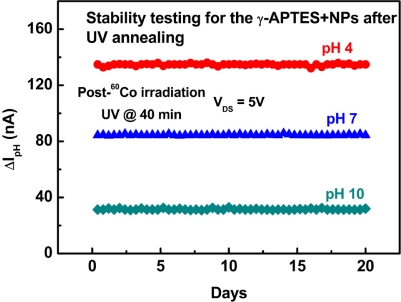
Consecutive pH tests for the γ-APTES+NPs/native-oxide/PSW pH-sensitive sensors with 40-min post-sterilization UV annealing.

Finally, we conducted a stability test for post-sterilization UV treated γ-APTES+NPs/native-oxide/PSW sensors. Figure 9 shows the results of daily pH analyses of pH 4, 7, and 10 solutions over three consecutive weeks for the γ-APTES+NPs/native-oxide/PSW sensors with post-sterilization UV annealing. We found that the post-sterilization pH-sensitive γ-APTES+NPs/native-oxide/PSW sensors show high stability in consecutive testing. We also conducted successive pH tests for each γ-APTES+NPs/native-oxide/PSW sensor following post-sterilization UV annealing. The relative standard deviation (RSD) of the 15 successive measurements for pH 4, 7, and 10 solutions are 5.57%, 5.32% and 5.65%, respectively. The same pH analyses for pH 4, 7, and 10 solutions were tested on three γ-APTES+NPs/native-oxide/PSW sensors from four different fabrication batches, with respective RSDs of 5.22%, 5.18%, and 5.42%, indicating the stability of the pH sensors following post-sterilization UV annealing.

## 4. Conclusions

Mixing PDMS-treated SiO_2_ NPs in γ-APTES with post-irradiation ultraviolet annealing can restore γ-ray sterilization-induced damage, resulting in stable sterilized pH-sensitive PSW sensors. It was found that trap charges induced by high-energy radiation were mostly concentrated in the native oxide layer for the γ-APTES-covered pH-sensitive PSW sensors, but accumulated in the γ-APTES+NPs nanocomposite layer for the γ-APTES+NPs-covered sensors. Variations of bright spots distribution over time in AFM surface morphology images are consistent with the change of the channel current change ΔI_DS_ following irradiation. Possible bond transformations and defect migrations to γ-APTES/SiO_2_ interfaces forming trap charges were expected. Given the wide use of γ-APTES as a material in biosensors, the detailed mechanism of γ-ray sterilization is worthy of further investigation.

## References

[1] Humenyuk I, Temple-Boyer P, Sarrabayrouse G (2008). The effect of γ-sterilization on the pH-ChemFET behavior. Sens. Actuat. A.

[2] Vaddirajua S, Tomazos I, Burgess DJ, Jain FC, Papadimitrakopoulos F (2010). Emerging synergy between nanotechnology and implantable biosensors: A review. Biosens. Bioelectron.

[3] Sadowski M, Gomez M, Moldenhauer J (2009). The use of biological indicators in the development and qualification of moist heat sterilization processes. Biological Indicators for Sterilization Processes.

[4] George LG, Vernon MG, David RS, Robert KH, Charles RP (1964). Effect of moisture on ethylene oxide sterilization. Appl. Environ. Microbiol.

[5] Suzuki H, Sugama A, Kojima N, Takei F, Ikegami K (1991). A miniature Clark-type oxygen electrode using a polyelectrolyte and its application as a glucose sensor. Biosens. Bioelectron.

[6] Graham GS, Mielnik TJ (1997). Industrial low-temperature gas plasma sterilization. Med. Device Technol.

[7] Yardimci O, Setlow P (2010). Plasma sterilization: Opportunities and microbial assessment strategies in medical device manufacturing. IEEE Trans. Plasma Sci.

[8] Moussy F, Harrison DJ, O’Brien DW, Rajotte RV (1994). *In vitro* and *in vivo* performance and lifetime of perfluorinated ionomer-coated glucose sensors after high-temperature curing. Anal. Chem.

[9] Herrmann S, Oelsner W, Kaden H, Brischwein M, Wolf B (2000). The influence of different methods of disinfection on the function of electrochemical sensors. Sens. Actuat. B.

[10] Iwaguch S, Matsumura K, Tokuoka Y, Wakui S, Kawashima N (2002). Sterilization system using microwave and UV light. Colloid. Surface. B.

[11] Abel PU, von Woedtke T (2002). Biosensors for *in vivo* glucose measurement: Can we cross the experimental stage. Biosens. Bioelectron.

[12] Batzer OF, Doty DM (1955). Radiation sterilization, nature of undesirable odors formed by gamma irradiation of beef. J. Agr. Food Chem.

[13] da Silva EF, Nishioka Y, Ma TP (1987). Radiation response of MOS capacitors containing fluorinated oxides. IEEE Trans. Nucl. Sci.

[14] Ma TP, Dressenforder PV (1989). Ionizing Radiation Effects in MOS Devices and Circuits.

[15] Listvan MA, Vold PJ, Arch DK (1987). Ionizing radiation hardness of GaAs technologies. IEEE Trans. Nucl. Sci.

[16] Markus H, John H, Sharp ID, Maren F, Martin S, Herwig GP, Stefan T (2010). Real-time x-ray response of biocompatible solution gate AlGaN/GaN high electron mobility transistor devices. Appl. Phys. Lett.

[17] Hsu PY, Lin JJ, Wu YL, Hung WC, Cullis AG (2009). Ultra-sensitive polysilicon wire glucose sensor using a 3-aminopropyltriethoxysilane and polydimethylsiloxane-treated hydrophobic fumed silica nanoparticle mixture as the sensing membrane. Sens. Actuat. B.

[18] Wu YL, Hsu PY, Lin JJ (2011). Polysilicon wire glucose sensor highly immune to interference. Biosens. Bioelectron.

[19] Lin JJ, Hsu PY, Wu YL, Jhuang JJ (2011). Characteristics of polysilicon wire glucose sensors with a surface modified by silica nanoparticles/γ-APTES nanocomposite. Sensors.

[20] Berdichevsky Y, Khandurina J, Guttman A, Lo YJ (2004). UV/ozone modification of poly(dimethylsiloxane) microfluidic channels. Sens. Actuat. B.

[21] Graubner VM, Jordan R, Nuyken O (2004). Photochemical Modification of Cross-Linked Poly(dimethylsiloxane) by Irradiation at 172 nm. Macromolecules.

[22] Wu YL, Lin JJ, Hsu PY, Hsu CP (2011). Highly sensitive polysilicon wire sensor for DNA detection using silica nanopartiles/γ-APTES nanocomposite for surface modification. Sens. Actuat. B Chem.

[23] Lin MC, Chu CJ, Tsai LC, Lin HY, Wu CS, Wu YP, Wu YN, Shieh DB, Su YW, Chen CD (2007). Control and detection of organosilane polarization on nanowire field-effect transistors. Nano Lett.

[24] Shalev G, Haopern E, Doron A, Cohen A, Rosenwaks Y, Levy I (2009). Surface chemical modification induces nanometer scale electron confinement in field effect device. J. Chem. Phys.

[25] Pena-Alonso R, Rubio J, Oteo JL (2007). Study of the hydrolysis and condensation of γ-Aminopropyltriethoxysilane by FT-IR spectroscopy. J. Mater. Sci.

[26] Pasternack RM, Amy SR, Chabal YJ (2008). Attachment of 3-(Aminopropyl)triethoxysilane on silicon oxide surfaces: dependence on solution temperature. Langmuir.

[27] Porti M, Nafryía M, Aymerich X, Cester A, Paccagnella A, Cimino S (2005). Irradiation induced weak spots in SiO_2_ gate oxides of MOS devices observed with C-AFM. Electron. Lett.

[28] Porti M, Nafryía M, Aymerich X, Cester A, Paccagnella A, Cimino S Leaky spots in irradiated SiO_2_ gate oxides observed with C-AFM.

